# Identifying microRNA determinants of human myelopoiesis

**DOI:** 10.1038/s41598-018-24203-7

**Published:** 2018-05-08

**Authors:** Megha Rajasekhar, Ulf Schmitz, Stephane Flamant, Justin J.-L. Wong, Charles G. Bailey, William Ritchie, Jeff Holst, John E. J. Rasko

**Affiliations:** 1 Gene & Stem Cell Therapy Program, Centenary Institute, University of Sydney, Camperdown, 2050 Australia; 2 Sydney Medical School, University of Sydney, Sydney, NSW 2006 Australia; 3 Gene Regulation in Cancer Laboratory, Centenary Institute, University of Sydney, Camperdown, 2050 Australia; 4 Origins of Cancer Program, Centenary Institute, University of Sydney, Camperdown, 2050 Australia; 5 Cell and Molecular Therapies, Royal Prince Alfred Hospital, Camperdown, 2050 Australia

## Abstract

Myelopoiesis involves differentiation of hematopoietic stem cells to cellular populations that are restricted in their self-renewal capacity, beginning with the common myeloid progenitor (CMP) and leading to mature cells including monocytes and granulocytes. This complex process is regulated by various extracellular and intracellular signals including microRNAs (miRNAs). We characterised the miRNA profile of human CD34^+^CD38^+^ myeloid progenitor cells, and mature monocytes and granulocytes isolated from cord blood using TaqMan Low Density Arrays. We identified 19 miRNAs that increased in both cell types relative to the CMP and 27 that decreased. miR-125b and miR-10a were decreased by 10-fold and 100-fold respectively in the mature cells. Using *in vitro* granulopoietic differentiation of human CD34^+^ cells we show that decreases in both miR-125b and miR-10a correlate with a loss of CD34 expression and gain of CD11b and CD15 expression. Candidate target mRNAs were identified by co-incident predictions between the miRanda algorithm and genes with increased expression during differentiation. Using luciferase assays we confirmed *MCL1* and *FUT4* as targets of miR-125b and the transcription factor *KLF4* as a target of miR-10a. Together, our data identify miRNAs with differential expression during myeloid development and reveal some relevant miRNA-target pairs that may contribute to physiological differentiation.

## Introduction

Myelopoiesis results in the generation of mature myeloid cells: the monocytes, granulocytes, dendritic cells, megakaryocytes and erythrocytes. Differentiation follows a highly regulated series of phases from a hematopoietic stem cell (HSC) into increasingly self-renewal- and lineage-restricted progenitor cells. Myelopoiesis begins from the common myeloid progenitor (CMP) that gives rise to the mature myeloid cells, but not to the T, B and NK cells of the lymphoid lineage.

The classical, mouse-based model of HSC differentiation, devised by the work of Akashi, Kondo and Weissman^1,2^ is relevant to human physiology as confirmed using cells from the bone marrow and umbilical cord blood and a specific set of CD markers^3–5^. In this model, long-term repopulating HSCs give rise to short-term HSCs, which in turn lead to multipotent progenitors and then the common lymphoid and myeloid progenitors. Human cells with the phenotype CD34^+^CD38^+^ have no self-renewing or lymphoid potential and are believed to constitute the CMP as well as the further differentiated granulocyte/monocyte progenitor (GMP), the megakaryocyte/erythroid progenitor (MEP) and a potential monocyte/dendritic cell progenitor (MDP). These subsets can be further differentiated based on expression of CD45RA and CD135^4^. Together these represent ~1% of the mononuclear cell population of the bone marrow^4,6^. While this hierarchy is generally widely accepted for human myelopoiesis^7,8^, further studies have refined the model by demonstrating a potential for macrophage and dendritic cell development from a lymphoid progenitor^9^ and by defining in exquisite detail several sub-types of dendritic cells and monocytes^10^.

Several extrinsic and intrinsic factors work in concert to determine the fate of stem and progenitor cells as they differentiate into mature cells. The primary extrinsic determinants of myeloid differentiation are the cytokines granulocyte/macrophage colony stimulating factor (GM-CSF), granulocyte colony stimulating factor (GCSF), macrophage colony stimulating factor (MCSF), erythropoietin (EPO), and thrombopoietin (TPO)^11^. The most influential intrinsic factors are believed to be a group of transcription factors, beginning with SCL/TAL-1 and RUNX1 in the embryo, followed by PU.1, C/EBPα, IRF8 and GFI1^12^. In addition to these, cell fate decisions may also be determined by other means including by post-transcriptional regulators exemplified by microRNAs (miRNAs).

MiRNAs are small, non-coding RNA molecules (19–22 nucleotides) that are genomically encoded and are involved in most aspects of cellular development and maintenance^13^. Initially transcribed as a long primary RNA, the transcript undergoes sequential cleavage by RNAse III family enzymes Drosha and Dicer to produce the mature miRNA which is incorporated into a protein complex named the RNA-induced silencing complex (RISC). The miRNA then guides the RISC to the 3′ untranslated regions (3′ UTRs) of messenger RNA (mRNA) molecules to which it binds while the RISC inhibits translation of the mRNA (reviewed in ref.^14^). Due to imperfect sequence complementarity each miRNA is predicted to bind hundreds to thousands of target mRNAs however, the degree of translational repression observed for each binding site is usually small^15^. Thus miRNAs are believed to impact specific phenotypes by broadly suppressing multiple targets within specific pathways rather than by large effects on a small number of targets^16^.

Determining the role of miRNAs in myelopoiesis has been approached from different angles previously, with the focus generally being on unilineage development or activation^17–19^ or on the transition from HSCs to committed progenitors^20–22^. Several of the reported studies have used cell lines such as NB4 or THP-1 differentiated *in vitro* using agents such as retinoic acid, which, it could be argued, may be a better model of myeloid malignancies rather than primary myelopoiesis^23–25^. Nonetheless, these studies have revealed interactions between key molecules in myelopoiesis and miRNAs. Myeloid transcription factors regulate expression of miRNAs and often form feedback loops e.g. C/EBP-α and miRs-34a, -223 and -30c^26–28^; Gfi1 and miRs-21 and -196b^29^; and several miRNAs regulated by PU.1^30^. Conversely, differential expression of several miRNAs such as miR-223 and miR-17–5p, and consequent regulation of their targets, is required for normal myelopoiesis^18,31^.

While the transition of HSCs to progenitors has been previously examined, few studies have directly evaluated the influence of miRNAs on cell fate decisions subsequent to the progenitor, particularly in the human context. More generally, studies have explored miRNAs in bulk human CD34^+^ cells, which would encompass both stem and progenitor cells, versus mature cells from the same source to identify lineage specific miRs^32,33^. One previous study by Lu *et al*. (2008) examined human bone marrow derived MEPs to identify miRNAs that influence cell fate choice between megakaryocytes or erythrocytes with the identification of miR-150 as crucial in this process^34^.

To date a comparable investigation during monocytopoiesis or granulopoiesis has not been reported. In this study we examined the miRNA profiles of primary myeloid progenitors, monocytes and granulocytes derived from human cord blood. We also examined the mRNA and miRNA profiles of the same cells in combination with target prediction software. Here we identified and validated targets of two miRNAs with relevance to human myelopoiesis.

## Results

### Isolation of myeloid populations

**Figure 1:**
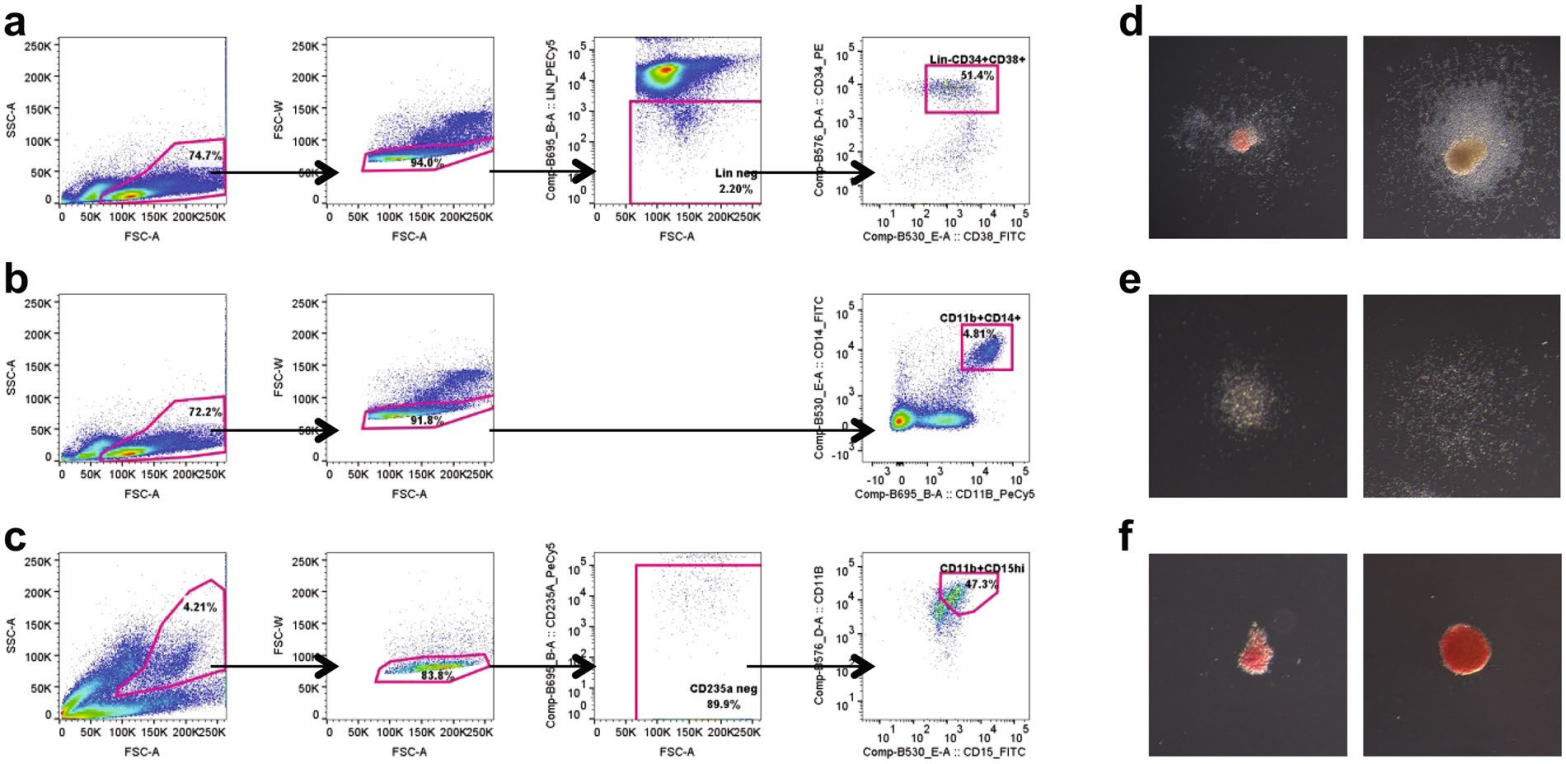
Purification of myeloid populations. (**a**) FACS gating strategy for purifying the CD34^+^CD38^+^ myeloid progenitor populations. Firstly, mononuclear cells were gated on, then single cells, followed by lineage-negative cells. (**b**) Monocytes were identified as the CD11b^+^CD14^+^ cells within cord blood mononuclear cells. (**c**) Polymorphonuclear cells were isolated by dextran separation from red blood cells. Granulocytes were then sorted and were identified as the CD11b^high^CD15^high^ cells, following exclusion of CD235a^+^ red blood cells. (**d**–**f**) Myeloid progenitors were plated in semi-solid Methocult medium with the cytokines SCF, IL-3, GM-CSF and EPO. Colony types formed include colony forming unit-granulocyte/erythrocyte/monocyte/macrophage (**d**, left), granulocyte/macrophage (**d**, right), granulocyte (**e**, left), macrophage (**e**, right), erythrocyte (**f**, left) and blast forming unit-erythrocyte (**f**, right). Note: Images **d**–**f** are on different scales to emphasize morphological features.

Myeloid progenitors and monocytes were isolated from human umbilical cord blood by fluorescence activated cell sorting (FACS), following the enrichment of mononuclear cells by density gradient centrifugation. We adopted the definition of myeloid progenitors as lin^−^CD34^+^CD38^+^ cells to incorporate the populations identified as the CMP, GMP and MEP. This conforms with classifications defined by Manz *et al*. (2002), based on colony forming potential and mRNA expression of relevant genes^4^. The myeloid progenitors in this study were purified using the same strategy, excluding mature cells with a lineage cocktail incorporating antibodies against the markers CD3, CD7, CD10, CD11b, CD14, CD19 and CD56, then sorting the CD34 and CD38 double positive cells (Fig. 1a). This lin^−^CD34^+^CD38^+^ population represents approximately 1% of the mononuclear cell fraction of cord blood. We further assessed their myeloid progenitor status using colony forming unit (CFU) assays in semi-solid Methocult medium supplemented with cytokines. Our results confirmed that the progenitor population isolated by us could give rise to CFU-granulocyte/erythrocyte/monocyte/macrophage (CFU-GEMM), CFU-granulocyte/macrophage (CFU-GM), CFU-granulocyte (CFU-G), CFU-macrophage (CFU-M), CFU-erythrocyte (CFU-E) and blast forming unit-erythrocyte (BFU-E) (Fig. 1d–f).

Expression of the integrin alpha M molecule (ITGAM), also known as CD11b, was used to identify monocytes and granulocytes in the enriched samples. Monocytes were identified as mononuclear cells with dual expression of CD11b and the bacterial lipopolysaccharide co-receptor CD14 (Fig. 1b). Granulocyte isolation was performed on the cells that separated from the mononuclear cells and settled with the red blood cells using a dextran gradient. During sorting granulocytes were identified following exclusion of contaminating red blood cells using CD235a and then isolating the cells positive for both CD11b and the granulocyte-specific carbohydrate adhesion molecule CD15 (Fig. 1c). The sorted cells were immediately stored for RNA extraction.

### MicroRNA profiling of myeloid cells

**Figure 2:**
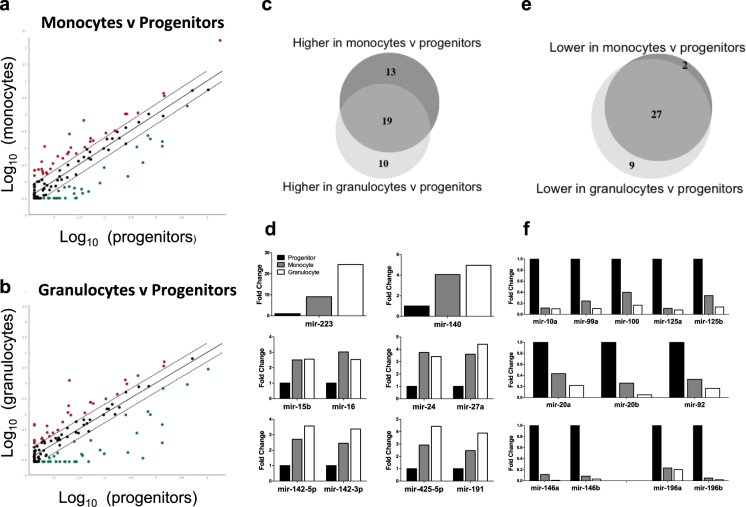
MiRNA profiling of cord blood myeloid progenitors, monocytes and neutrophils. Normalised TaqMan Low-Density Array (TLDA) profiles of miRNA expression in (**a**) monocytes vs. myeloid progenitors and (**b**) granulocytes vs. myeloid progenitors from human cord blood. MiRNAs with ≥ 2-fold increased expression in the mature cell types are shown in red and those with ≥ 2-fold decreased expression are shown in green. (**c**) Number of miRNAs increased ≥ 2-fold or more in each comparison. (**d**) Top panel: miRNAs with a ≥ 2-fold increase in both cell types. Middle and lower panel: miRNAs that are increased in both cell types and are clustered together in the genome. (**e**) Number of miRNAs decreased ≥ 2-fold in each comparison. (**f**) Top panel: the five miRNAs of the same family (highly similar seeds) that are decreased in both mature cell types relative to the progenitor. Middle and lower panels: miRNAs that decreased in both comparisons and are clustered together in the genome.

Next, TaqMan Low Density Arrays (TLDAs) were used to profile the expression of 365 miRNAs, of which 113 were amplified in at least one of the three cell types. In a pairwise comparison of gene expression in progenitors vs. monocytes 33 miRNAs were increased in monocytes, while 29 miRNAs were reduced (≥2-fold) (Fig. 2a and Table 1). When comparing progenitors with granulocytes, 30 miRNAs were increased in granulocytes and 36 miRNAs were reduced (≥2-fold) (Fig. 2b and Table 1).

**Table 1:** MicroRNAs that are differentially expressed by at least 2-fold between myeloid progenitors and corresponding monocytes and granulocytes.

Increased in monocytes and granulocytes vs. progenitors		Decreased in monocytes and granulocytes vs. progenitors	
miR-223		let-7c	miR-222
miR-328		miR-100	miR-296
miR-103		miR-10a	miR-335
miR-140		miR-125a	miR-372
let-7b		miR-125b	miR-486
miR-24		miR-126-5p	miR-545
miR-197		miR-126-3p	miR-92
miR-27a		miR-127	miR-99a
miR-301		miR-130a	
miR-374		miR-135a	
miR-16		miR-146a	
miR-425-5p		miR-146b	
miR-142-5p		miR-155	
miR-484		miR-181d	
miR-26b		miR-196a	
miR-15b		miR-196b	
miR-191		miR-203	
miR-142-3p		miR-20a	
miR-199a		miR-20b	
**Increased in monocytes only**		**Decreased in monocytes only**	
miR-21	miR-15a	miR-101	
miR-532	miR-362	miR-425	
miR-340	let-7g		
miR-660	miR-195		
miR-28	miR-342		
miR-324-5p			
**Increased in granulocytes only**		**Decreased in granulocytes only**	
miR-145	miR-361	let-7d	
miR-148a	miR-365	miR-152	
miR-192	miR-517c	miR-17-5p	
miR-210		miR-18a	
miR-29a		miR-19a	
miR-30b		miR-19b	
miR-32		miR-320	
**Increased in monocytes and decreased in granulocytes**			
miR-339			
miR-151			

In order to identify miRNAs that are increased with differentiation in general, we combined expression data from monocytes and granulocytes. A subset of 19 miRNAs were increased in both monocytes and granulocytes compared to progenitors (Fig. 2c and Table 1). miR-223 showed the greatest increase, with levels in granulocytes and monocytes 24- and 9-fold higher than in progenitors (Fig. 2d, top panel). Among the commonly increased candidates were also several miRNAs that are clustered together in the genome and are often expressed in a similar pattern as each other (Fig. 2d, middle and lower panels). Interestingly, a similar comparison of commonly down-regulated genes revealed 27 miRNAs that were decreased in both monocytes and granulocytes relative to progenitors (Fig. 2e). These include five of the eight members of the miR-10 gene family (miRs-10a, -99a, -100, -125a and -125b, as classified by miRBase) (Fig. 2f, top panel) and also several clustered miRNAs (miRs-20a, -20b and -92, Fig. 2f middle and lower panels).

**Figure 3:**
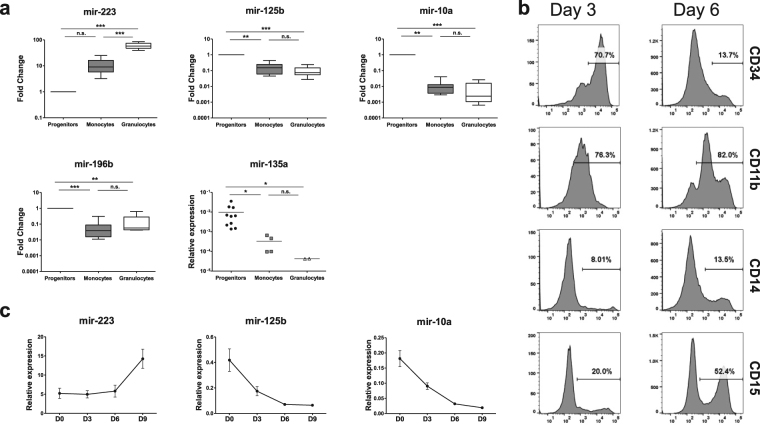
Decreased expression of miR-125b and miR-10a is associated with increased lineage commitment of myeloid progenitors. (**a**) qRT-PCR validation of miRNAs differentially expressed in TLDA experiments (n = 10). The small nucleolar RNA RNU24 was used for normalisation. Whiskers show 5th and 95th percentiles. miR-135a could not be amplified from most monocyte and neutrophil samples; the results have therefore been shown here as expression relative to RNU24 only. Differences were tested by ANOVA, Kruskal Wallis test with Dunn’s post test. ***p < 0.001, **p < 0.01, *p < 0.05, n.s. = not significant. (**b**) Expression of CD34, CD11b, CD14 and CD15 on MACS-purified cord blood CD34^+^ cells cultured for 6 days with SCF (100 ng/mL) and GCSF (10 ng/mL). (**c**) The same cytokines were used to *in vitro* differentiate FACS-purified CD34^+^ cells and their levels of miR-223, miR-125b and miR-10a assessed by q-RT-PCR over nine days (n = 3). Graphs show mean ± SEM.

Five differentially expressed miRNAs were chosen for qRT-PCR validation, with expression normalised to a snoRNA RNU24 (Fig. 3a). miR-223, miR-125b, miR-10a, miR-196b and miR-135a showed similar patterns of expression to that observed in the TLDA data. When measured by qRT-PCR the mean level of miR-223 was ten-fold higher in monocytes relative to progenitors, and was sixty-fold higher in granulocytes. miR-125b was expressed at ten-fold lower levels in monocytes and granulocytes relative to progenitors, consistent with the TLDA results. The Hox-cluster encoded miR-10a could be amplified from all the progenitor samples but only from seven monocyte samples and eight granulocyte samples. Mean expression of miR-10a was a hundred-fold lower in both monocytes and granulocytes, making the levels too low to be detected from the remaining five monocyte and granulocyte samples.

The second Hox-cluster encoded miRNA, miR-196b, was expressed at thirty-fold lower levels in monocytes and fifteen-fold lower levels in granulocytes, relative to progenitors. miR-135a could be detected in all the progenitor samples, though the levels of miRNA were quite low. It could only be amplified from four monocyte samples and two granulocyte samples and showed approximately thirty-fold lower levels in the mature cells relative to the progenitor cells.

Two other studies have reported the miRNA expression profiles of differentiating hematopoietic stem and progenitor cells. Bissels *et al*. (2011) compared bone marrow CD133^+^ (more primitive) with CD133^−^CD34^+^ cells (less primitive)^35^ while Liao *et al*. (2008) studied more differentiated cells and compared CD34^+^CD38^−^ cells with CD34^+^ cells^36^. Similarly, Cattaneo *et al*. (2015) compared miRNA profiles of CD34+CD38− cells (HSCs) and CD34+CD38+ cells (hematopoietic progenitor cells; HPCs)^20^. The miRNA profiles of the progenitors in our study are comparable as they could be placed at the next step with their more differentiated phenotype of CD34^+^CD38^+^. The miRNA profiles in these three studies and in this study were obtained using four different profiling platforms and therefore vary in the miRNAs included. Nonetheless, in order to identify miRNAs that are increased or decreased with differentiation we compared those that were reported as increasing or decreasing with differentiation in these three studies with those in ours.

We found ten miRNAs that decrease with differentiation in at least three of these four studies, suggesting that they may be involved in maintenance of a more undifferentiated phenotype and seven miRNAs that increase (Table 2). Interestingly, four of the reduced miRNAs, miR-99a, miR-100, miR-125b, miR-125a-5p, are members of homologous tricistronic clusters involved in stem and progenitor cell homeostasis^37^. Therefore there are 12 miRNAs that increase and 18 that decrease with differentiation from this study that were not reported by the previous studies of stem cells/progenitors. The miRNAs that increase with differentiation in our study may indicate some that are required for a terminally differentiated phenotype (e.g. miR-223 for granulocytes and miR-15b for megakaryocytes) and the ones that decrease tend to be those that help to maintain the stem/progenitor cell phenotype (e.g. let-7c).

**Table 2:** Stem/progenitor microRNAs that consistently increase and decrease during differentiation as demonstrated by Bissels *et al*.^35^, Liao *et al*.^36^, Cattaneo *et al*.^20^ and this study.

Increased in Bissels *et al*. and this study	Increased in Liao *et al*. and this study	Increased in Cattaneo *et al*. and this study	Commonly increased in three studies
miR-484	miR-16	*nil*	miR-142-3p
miR-425-5p	miR-27a		miR-142-5p
miR-191			
**Decreased in Bissels** ***et al***. **and this study**	**Decreased in Liao** ***et al***. **and this study**	**Decreased in Cattaneo** ***et al***. **and this study**	**Decreased in Bissels** ***et al***. **and Cattaneo** ***et al***.
miR-146a	miR-127	miR-126-5p	miR-29b-3p
miR-146b-5p	miR-100		
miR-99a			
miR-10a			
miR-125b			
miR-125a-5p			

These data together suggest a signature of miRNA expression associated with differentiation status and maturation within the myeloid lineage. They also provide some evidence of monocyte and granulocyte lineage specific miRNAs with varying expression in each of the mature populations.

### Decreased expression of miR-10 family members is associated with increased lineage commitment

The miRBase database lists miR-s-10a, -10b, -125a, -125b, -99a, -99b and -100 as members of the miR-10 family. As there was a clear association of decreased expression of these miRNAs with more mature phenotypes, we further explored two family members, miR-125b and miR-10a in this context.

In initial screening experiments we sought to explore the change in levels of these miRNAs in response to short-term liquid culture. FACS purified cord blood CD34^+^ cells were cultured in medium with serum and SCF or IL-3 (Supplementary Figure 1). Cells were sampled at days 1, 2 and 3 and levels of miR-125b and miR-10a assessed by qRT-PCR. In response to SCF, levels of miR-125b decreased by an average of 40% by day 3. In response to IL-3, miR-125b levels decreased by an average of 60%. Levels of miR-10a mirrored this and decreased 50% by day 3 with SCF and 80% with IL-3.

While SCF and IL-3 by themselves can stimulate granulocyte/macrophage colony formation, the combination of SCF with GCSF is a more potent and specific inducer of myeloid commitment in CD34^+^ cells in liquid and semi-solid cultures^38^. In order to focus on granulocytic commitment using longer culture duration, MACS-purified cord blood CD34^+^ cells were differentiated *in vitro* using SCF and GCSF and their cell-surface expression of CD34, CD11b, CD14 and CD15 assessed. By day 6, expression of CD34 was decreased from approximately 70% to 15% of cells, with a concomitant increase in surface expression of the mature myeloid markers CD11b, CD14 and particularly of CD15 (Fig. 3b). Over a similar time course qRT-PCR showed a seven-fold decrease in miR-125b and approximately ten-fold decrease in miR-10a by day 9 (Fig. 3c). miR-223 expression was used as a positive control and showed an approximately three-fold increase by day 9 (Fig. 3c). These data confirm the decrease in expression of miR-10a and miR-125b during early myeloid differentiation.

### Identification of predicted targets of miR-125b and miR-10a

**Figure 4:**
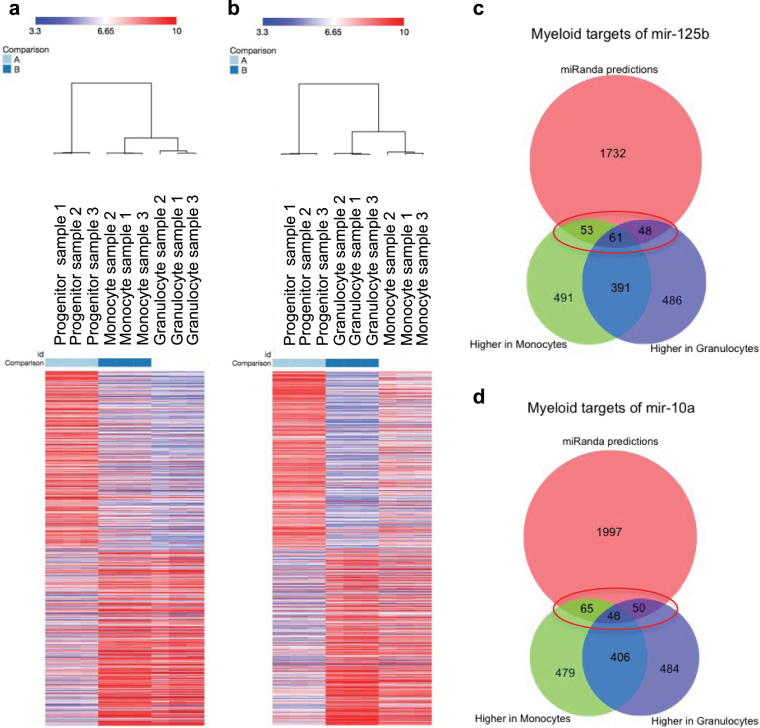
Identifying miRNA targets. Gene expression profiling was performed on progenitors, monocytes and granulocytes isolated from three human cord blood samples (1,2,3) using Affymetrix microarrays. The heatmaps show levels (log 2) of the top 2000 differentially expressed genes between (**a**) progenitors and monocytes and (**b**) progenitors and granulocytes. The identity of transcripts exhibiting higher expression in the mature cell types relative to progenitors was combined with target predictions for miR-125b (**c**) and miR-10a (**d**), both miRNAs with lower expression in the mature cells. The resulting 162 and 161 genes respectively (circled in red in c and d) that had inverse expression with the miRNA as well as being predicted targets were analysed further.

Affymetrix microarrays were performed on progenitors, monocytes and granulocytes that had been FACS isolated as described above from three cord blood samples. Unsupervised hierarchical clustering showed that the gene expression of the two mature cell types was closer to each other than to the progenitors (Supplementary Figure 2). To assess the differences in gene expression between the progenitors and each of the mature populations, we examined the top 2000 genes that were increased and decreased in monocytes vs. progenitors and in granulocytes vs. progenitors (Fig. 4a and b). Gene ontology analysis of these sets showed that the differentially expressed genes were enriched in pathways pertaining to the acquisition of features of mature myeloid cells (Table 3). In the comparison of progenitors with mature monocytes the most significantly over-represented pathways include ‘regulation of myeloid leukocyte mediated immunity’, ‘toll-like receptor signalling pathway’ and ‘pattern recognition receptor signalling pathway’. In progenitors versus granulocytes the most significantly over-represented pathways included ‘granulocyte activation’, ‘rRNA processing’ and ‘nonsense-mediated decay’. These results further confirm the identity and maturation status of the cells used in our study.

**Table 3:** The top 15 categories from gene ontology analysis performed on the top 2000 differentially expressed transcripts using the Panther database’s statistical over representation test with Bonferroni correction (www.pantherdb.org).

GO biological process complete	Fold enrichment	p-value
**Progenitors vs**. **Monocytes**		
regulation of mast cell activation (GO:0033003)	3.98	4.22E-02
regulation of myeloid leukocyte mediated immunity (GO:0002886)	3.98	4.22E-02
regulation of B cell proliferation (GO:0030888)	3.48	2.19E-02
regulation of alpha-beta T cell activation (GO:0046634)	3.30	5.91E-03
toll-like receptor signaling pathway (GO:0002224)	3.00	1.84E-02
purine ribonucleoside biosynthetic process (GO:0046129)	2.95	1.56E-02
purine nucleoside biosynthetic process (GO:0042451)	2.95	1.56E-02
nucleoside biosynthetic process (GO:0009163)	2.94	5.12E-04
pattern recognition receptor signaling pathway (GO:0002221)	2.92	2.17E-03
glycosyl compound biosynthetic process (GO:1901659)	2.89	7.45E-04
purine nucleotide biosynthetic process (GO:0006164)	2.89	2.12E-04
positive regulation of lymphocyte proliferation (GO:0050671)	2.88	1.48E-04
positive regulation of mononuclear cell proliferation (GO:0032946)	2.84	2.14E-04
ribonucleoside biosynthetic process (GO:0042455)	2.82	6.51E-03
positive regulation of leukocyte proliferation (GO:0070665)	2.81	1.79E-04
**Progenitors vs**. **Granulocytes**		
maturation of LSU-rRNA (GO:0000470)	6.41	2.42E-04
neutrophil activation (GO:0042119)	5.87	3.73E-02
granulocyte activation (GO:0036230)	5.82	1.51E-02
ribosomal large subunit biogenesis (GO:0042273)	3.88	3.31E-04
SRP-dependent cotranslational protein targeting to membrane (GO:0006614)	3.34	2.03E-04
myeloid leukocyte activation (GO:0002274)	3.32	4.57E-05
viral gene expression (GO:0019080)	3.14	2.57E-05
cotranslational protein targeting to membrane (GO:0006613)	3.08	1.11E-03
protein targeting to ER (GO:0045047)	3.08	1.11E-03
rRNA metabolic process (GO:0016072)	3.03	5.71E-13
rRNA processing (GO:0006364)	3.03	1.45E-12
nuclear-transcribed mRNA catabolic process, nonsense-mediated decay (GO:0000184)	3.03	2.55E-04
mitochondrial translational elongation (GO:0070125)	3.02	2.71E-02
ribosome biogenesis (GO:0042254)	2.99	1.45E-15
translational termination (GO:0006415)	2.99	1.29E-02

In order to identify mRNA targets of our miRNAs of interest from among these differentially expressed genes, we first considered bioinformatically-predicted targets of the miRNAs using the miRanda algorithm^39,40^, which uses sequence composition, conservation and thermodynamic stability as criteria for predicting miRNA target sites. This algorithm applies a sensitive method that has a high relative overlap with predictions from other algorithms. The top 1000 gene candidates that were increased in monocytes and granulocytes versus myeloid progenitors were combined with the target predictions for miR-125b and miR-10a (Fig. 4c and d). The resulting 162 candidates for miR-125b and 161 candidates for miR-10a that were both predicted targets and had inverse expression patterns to the miRNAs were investigated further through literature searches. This process resulted in the selection of three putative targets for confirmatory testing using the luciferase reporter assay (*MCL1*, *FUT4* and *KLF4*). The increase in *MCL1*, *FUT4* and *KLF4* expression during myeloid differentiation as determined in the microarray analysis is shown in Supplementary Figure 3.

### Experimental confirmation of targets of miR-125b and miR-10a by luciferase assays

**Figure 5:**
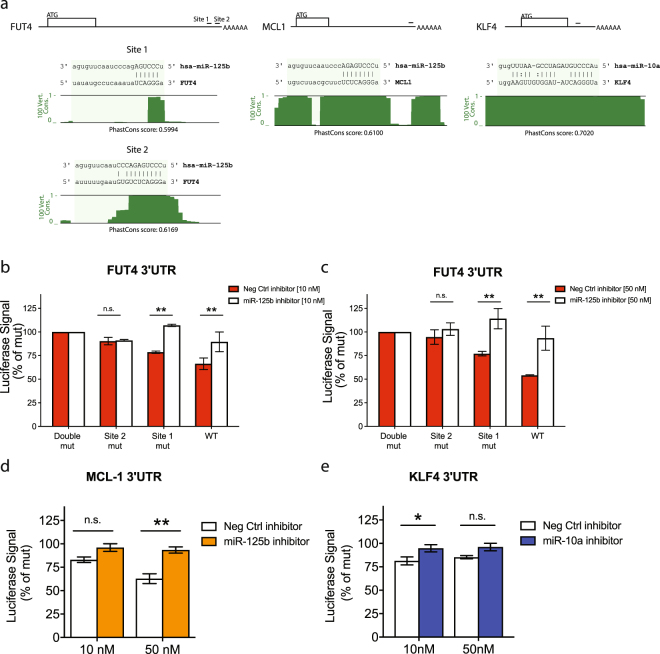
Gene targets of miR-125b and miR-10a. Luciferase reporter assays performed in HeLa cells transfected with the WT or mutated 3′ UTR binding site from (**a**) *MCL1*, (**b**,**c**) *FUT4* or (**d**) *KLF4*. The 3′ UTRs were cloned into a pSiCHECK2 vector and transfected with or without a specific inhibitor of mir-125b/ miR-10a or a non-specific control at 10 nM or 50 nM. The one predicted binding site in the *MCL1* and *KLF4* 3′ UTRs were tested, while the two binding sites in *FUT4* were each tested alone (Site 1 mutant and Site 2 mutant) and together (Double mutant). Each experiment was performed 3 times, each in triplicate. Graphs show mean ± SEM. Groups were tested by a two-way ANOVA with Bonferroni post tests. **p ≤ 0.01, * *p* ≤ 0.05, n.s. = not significant.

*FUT4* mRNA has two predicted miR-125b binding sites (~100 nt apart) in its 3′ UTR, whilst *MCL1* mRNA also contains a predicted miR-125b target site in its 3′ UTR (Fig. 5a). PhastCons scores for *FUT4* and *MCL1* miR-125b binding sites indicate that their seed regions are highly conserved in vertebrates (Fig. 5a). *KLF4* mRNA contains a predicted miR-10a binding site, which is also highly conserved in vertebrate species (Fig. 5a). To experimentally validate these miRNA-target interactions we used HeLa cells in which we had confirmed the presence of endogenously expressed miR-125b and miR-10a by qRT-PCR (Supplementary Figure 4). HeLa cells were transfected with luciferase reporter vectors carrying either WT or mutated (mut) 3′ UTRs of interest located downstream of luciferase. If repression of the WT vector relative to the mut vector was observed, they were then co-transfected with miR-125b or miR-10a inhibitor (125I/10I) or negative control (NC) inhibitor to knock down the miRNA expression and ‘rescue’ the repression.

As the 3′ UTR of *FUT4* has two predicted binding sites for miR-125b (Fig. 5a), repression of the WT 3′ UTR was tested alongside single mutants of each of the binding sites (Site 1 mut and Site 2 mut), and a construct with both seed regions disrupted (double mut). At the lower inhibitor concentration of 10 nM the luciferase signal from the WT vector with NC was only 66% of the double mut vector indicating repression by cell intrinsic miR-125b (Fig. 5b). This recovered to 90% when the WT vector was co-transfected with 10 nM 125I (p < 0.05). Upon mutation of miR-125b Site 1 in *FUT4*, the luciferase signal decreased to 79% of the double mut vector (i.e. 21% lower signal due to miR-125b binding the intact Site 2). This Site 1 mut vector was completely de-repressed when co-transfected with 125I (p < 0.05). Conversely, the vector containing the mutant Site 2 had a luciferase signal that was 90% of the double mut vector, i.e. only a modest 10% signal decrease due to miR-125b-mediated repression through the intact Site 1. Upon addition of 125I to the Site 2 mutant construct no de-repression was observed, confirming that minimal repression occurred through binding Site 1.

A similar pattern was observed when the same experiment was repeated at the higher concentration of the miRNA inhibitors. At 50 nM, the *FUT4* WT vector was de-repressed by 39% with 125I vs. double mut (Fig. 5c, p < 0.01) and the Site 1 mut vector was de-repressed by 37% with 125I (p < 0.05), as a result of the effect of miR-125b/125I on the intact, conserved binding Site 2 in this vector. As at the lower inhibitor dose, there was no significant difference in luciferase expression in the Site 2 mut vector between NC or 125I transfected samples, due to minimal repression by miR-125b binding to the intact site 1 in this vector. These data together suggest that *FUT4* expression is modulated by miR-125b by repression of the transcript through two binding sites, with most of the repression due to the more conserved of the two sites.

Luciferase vectors carrying either the WT or mut *MCL1* 3′ UTR were co-transfected with NC or 125I inhibitors at 10 nM or 50 nM. At 10 nM NC inhibitor, the luciferase signal of the WT vector was only 83% of the mut vector (Fig. 5d) indicating repression by miR-125b. When co-transfected with 125I the WT signal recovered to 96% of the mut, though this was not significant. At the higher inhibitor concentration of 50 nM a small non-specific inhibition was observed (31% in WT + NC vs. 10 nM and 35% in mut + 125I vs. 10 nM), however de-repression as a result of specific inhibition of miR-125b was still observed. At 50 nM, the WT luc signal was 63% of mut with NC, but recovered to 93% upon addition of 125I (p < 0.01), suggesting that miR-125b does regulate the expression of *MCL1*.

The *KLF4* gene has one predicted binding site for miR-10a in its 3′ UTR. Luciferase assays comparing the 3′ UTR with intact (WT) or mutated (mut) versions of this binding site showed modest but consistent and significant repression through this binding site (Fig. 5e). Repression could be recovered upon addition of 10I (14% de-repression at 10 nM, p < 0.05; 11% de-repression at 50 nM). The dose of the inhibitor did not affect the degree of de-repression.

In summary, luciferase reporter assays confirmed both *MCL1* and *FUT4* as direct targets of miR-125b and *KLF4* as a target of miR-10a.

## Discussion

MicroRNAs are crucial regulators of differentiation and lineage enforcement in the hematopoietic system^41^. While studies have examined miRNA profiles of bulk CD34^+^ cells, no study has specifically examined myeloid progenitors and their progeny concurrently. This study presents a miRNA profile of human myeloid progenitors and corresponding mature monocytes and granulocytes performed on highly pure and clearly defined primary cells. We identified miRNAs that are commonly increased and decreased upon differentiation in both lineages and also those that are unique to each lineage. For example, we demonstrate the pattern of expression of miR-125b in primary human cells, whereas previous data were obtained using murine cells. We also identified and experimentally validated some novel targets of miR-125b and another myeloid-relevant miRNA, miR-10a in this particular cellular context.

The role of miRNAs in myeloid leukaemia has been reported extensively^42^, however close examination of their role in primary human non-leukaemic stem and progenitor cells has been relatively limited. We observed a consistent pattern in the miRNA profile of our progenitor cells, and those previously reported in CD34^+^ cells^21,32^. This adds validation to the miRNAs generally identified as stem/progenitor-expressed (Table 2). In addition to this our data have highlighted human hematopoietic differentiation associated miRNAs (Table 1).

While studies by Bissels *et al*., Liao *et al*. and Cattaneo *et al*. focused on identifying miRNAs in more primitive stem cells^20,35,36^ our data identify miRNA changes after the cells pass these early stages and move towards maturity. The importance of the miRNAs we have identified in this manner are highlighted by the roles of some of them in other models. For instance, of the miRNAs that decrease with hematopoietic differentiation in our analysis, mouse studies have shown that a decrease in miR-146a is crucial for megakaryocytopoiesis^43^ and a decrease in the miR-99a/miR-125a cluster is important for loss of stemness as the cells mature^44^. Similarly, an increase in miR-142-3p is necessary for differentiation of myeloid cell lines^45^ and an increase in miR-16 is necessary for erythropoiesis^46^. Several of the miRNAs identified by us as relevant for monocyte or granulocyte differentiation have also been reported in *in vitro* models of human myeloid differentiation^22,33^.

**Figure 6:**
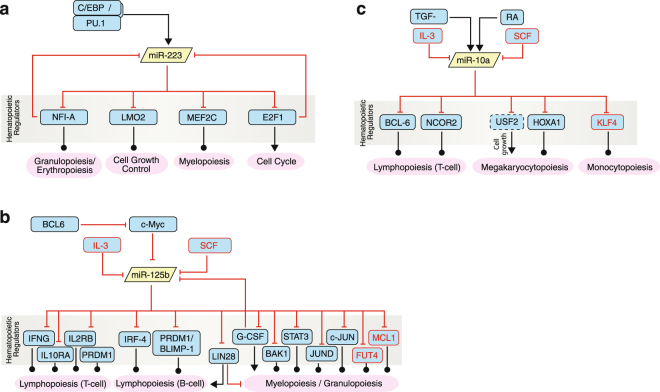
The miR-223, miR-125b and mir-10a regulatory circuits in human hematopoiesis. miR-223 regulates myeloid differentiation via the transcription factors LMO, MEF2C, NFI-A, and E2F1. The latter two are themselves negative regulators of miR-223 expression and thus fine-tune miR-223 in a negative feedback loop (**a**). miR-125b is involved in the regulation of T-cell and B-cell differentiation as well as granulopoiesis by repressing the expression of a number of different transcription factors. We found that miR-125b expression is inhibited by IL-3 and SCF, among other regulators (**b**). miR-10a is another regulator of myeloid and lymphoid differentiation targeting BCL-6 and NCOR2 in lymphopoiesis, USF2 and HOXA1 in megakaryocytopoiesis, and KLF4 in monocytopoiesis.

The upregulation of miR-223 we observed during monocytopoiesis and granulopoiesis, has also been recently shown^31^, but with their additional observation of miR-223 being maintained at low levels during erythropoiesis. The authors further show that the lineage-specific expression levels of miR-223 are fine-tuned by the coordinated recruitment and function of different lineage-specific transcription factors to the miR-223 regulatory regions^31^. The functional role of miR-223 in granulopoiesis is realized through a regulatory circuit involving the transcription factors C/EBPβ, PU.1, NFI-A and C/EBPα^47^. miR-223 itself represses the expression of NFI-A and other transcription factors that are important in hematopoietic differentiation processes such as MEF2C, a transcription factor that promotes myeloid progenitor proliferation^48^, LMO2, a key regulator of erythropoiesis^49^, and E2F1, the master regulator of cell-cycle progression^27^. An overview summarising the miR-223 regulatory circuitry in myeloid differentiation is illustrated in Fig. 6a.

In addition to these miRNAs that have been previously reported, we have also identified some miRNAs that have not been studied in the context of myeloid differentiation, such as miR-127 and miR-484. Both of these miRNAs have roles defined predominantly in cancer^50–52^ and may provide future opportunities for further investigation.

The cytokines SCF and IL-3 are often used in combination and with other cytokines for *ex vivo* expansion of hematopoietic stem cells^53^. Prior to our experiments in primary CD34^+^ cells, we tested the short-term effects of these cytokines on levels of miR-125b and miR-10a and found them to cause a relatively modest decrease in their levels. These findings correlate well with previous reports of these cytokines having a similar modest effect on granulocyte/macrophage colony formation in semi-solid and liquid cultures^38^. To delve deeper into lineage commitment and the role of our miRNAs of interest, we focused on granulopoiesis and extended the culture for longer. Cytokine mediated *in vitro* differentiation of progenitors towards the granulocytic phenotype with GCSF (and SCF) resulted in the decrease of both miR-125b and miR-10a to a much greater degree (approx. 10-fold), and correlated well with acquisition of differentiation markers over the same period.

Several studies in the mouse have identified miR-125b and family member miR-125a as important for various aspects of primitive and leukaemic hemopoiesis^54^. In terms of the myeloid lineage, the earliest report by Bousquet *et al*. showed that a common translocation in AML results in miR-125b overexpression^55^. Overexpression of miR-125b resulted in various leukaemias in mice^56^. In fact, several groups have over-expressed miR-125b in the mouse hematopoietic system using different methods^56–58^ and have all observed various myeloproliferative disorders depending on the method and degree of overexpression. Together these studies also report that the highest level of miR-125b is found in long-term HSCs, with levels progressively decreasing in more differentiated progenitors. The important roles of miR-125b and miR-125a in human myelopoiesis is highlighted by their frequent dysregulation in various myeloid malignancies (reviewed in ref.^54^). However, little has been reported regarding miR-125b in lineage definition of primary cells such as granulocytes.

In mouse, levels of miR-125b (and miR-125a) decrease with increasing granulocyte maturity^59^. In order to understand dynamic changes, two studies have examined the role of miR-125b in the murine IL-3 dependent promyelocytic cell line 32D^60,61^. Both studies report that 32D cells have very low levels of miR-125b, but Surdziel *et al*. found that miR-125b levels increased upon GCSF-induced differentiation^61^. Our experiments, performed with primary human cells found the contrary; that miR-125b levels decreased during granulocytic differentiation with GCSF, agreeing with findings in primary murine granulocytes^59^. Our findings are further supported by two studies involving miRNA expression at different stages of human neutrophil maturation^19,62^. Surdziel *et al*. and Bousquet *et al*. observed that over-expression of miR-125b in murine 32D cells resulted in increased proliferation and maintenance of an immature blast cell phenotype^60,61^. These findings, coupled with our observation that miR-125b levels decrease with increasing maturity, suggest that reduced miR-125b is necessary for normal (i.e. non-dysplastic) granulocyte development.

We anticipate that miR-125b achieves its regulatory effects through repression of target mRNA molecules. MiRNA target prediction algorithms use various parameters including degree of complementarity with target mRNA site, miRNA and binding site conservation and number of sites per mRNA to make their predictions across all transcripts^63^. In order to identify transcripts of relevance, we validated transcripts that were both predicted targets and expressed.

The first miR-125b target we validated was *FUT4*. *FUT4* encodes a member of the fucosyltransferase family, that produce carbohydrate moieties on the surface of cells that act as ligands for selectins and facilitate granulocyte adhesion to, and rolling on, endothelial cells. FUT4 is responsible for fucosylating the CD15 cell surface antigen in monocytes and promyelocytes^64^, as well as the production of ligands for P- and L- selectin in conjunction with another fucosyltransferase, FUT7^65,66^. The levels of *FUT4* (and *FUT7*) transcripts increase in response to GCSF, along with E-selectin ligands on immature and late mature granulocytes, as well as levels of E-selectin on endothelium^67^. These reports combined with our findings that *FUT4* is a target of miR-125b also highlight an important relationship between GCSF, miR-125b and FUT4 in the development and function of myeloid cells. Interestingly, *FUT7* which encodes a similar enzyme expressed in myeloid cells, also has a predicted binding site for miR-125b in its 3′ UTR, though this was not experimentally validated. These observations add further evidence that decreasing expression of miR-125b during myeloid differentiation contributes to the development of various lineage-specific and lineage-defining features.

The second validated miR-125b target was *MCL-1*, which encodes an anti-apoptotic member of the Bcl-2 family. A regulatory relationship between this miRNA/mRNA pair has been reported in various cancers^68–70^ but not in a myeloid context, where MCL-1 plays an important role. Conditional knock-out mice lacking *Mcl-1* in neutrophils and macrophages develop with no neutrophils and severely depleted monocytes^71,72^. These mice have increased numbers of immature myeloid precursors in the bone marrow. The administration of GCSF did not lead to increased peripheral blood granulocytes, suggesting that this process requires MCL1^72^. Our findings that miR-125b expression is decreased in response to GCSF and that miR-125b represses the mRNA for MCL1 suggests a regulatory relationship between this miRNA/mRNA pair and the process of GCSF-mediated granulopoiesis.

In summary, miR-125b targets are central to regulating important organismal processes including apoptosis, innate immunity, inflammation and hematopoietic differentiation^54^. During hematopoietic differentiation, miR-125b regulates a number of genes including cytokines and transcription factors critical for lymphoid and myeloid development (summarised in Fig. 6b). These include transcription factors BLIMP-1 and IRF-4 during human B-lineage differentiation^54^ and IFNG, IL2RB, IL10RA and PRDM1 during T-cell differentiation^73^. miR-125b also represses the expression of the RNA-binding protein Lin-28 which has been shown to be an important regulator of hematopoietic development^74^. In myelopoiesis, miR-125b is both induced by GCSF and negatively regulates GCSF expression via a feedback loop^54^ and also targets transcription factors STAT3, c-JUN and JUND and the pro-apoptotic effector BAK1^61^. We have now shown that the miR-125b regulatory circuitry is de-repressed during myeloid differentiation leading to an upregulation of FUT4 and MCL1 (Fig. 6b).

The second miRNA examined in this study, miR-10a, also exhibits decreasing expression during differentiation. Previously, a decrease in miR-10a has been reported during megakaryocytopoiesis^75^ and knock down of miR-10a in CD133^+^ progenitors can induce megakaryocytic differentiation via the transcription factor HOX1A^76^. An outstanding potential target of this miRNA was the transcription factor KLF4. While KLF4 is best known as one of the four factors necessary for reprogramming somatic cells into induced pluripotent stem cells, KLF4 is also a crucial transcription factor for monocyte development from CMPs, acting downstream of PU.1^77^. While the targeting of *KLF4* by miR-10a has been previously suggested to be relevant in AML^78^, we have shown that the decrease in miR-10a expression upon primary myeloid differentiation and the consequent release of repression may be necessary for monocytopoiesis (Fig. 6c).

Other hematopoietic regulators that are targets of miR-10a repression include BCL-6 and NCOR2, transcriptional repressors involved in T cell differentiation^79^. In lymphopoiesis miR-10a expression is induced by retinoic acid and TGFß^79^. Apart from the validated target HOX1A, qRT-PCR-based target validation suggests the cell growth inducer USF2 as another target of miR-10a important in megakaryocytopoiesis^80^.

In summary, by examining primary human cells at defined points in myeloid differentiation we have identified miRNAs that direct specific targets, with potential for great impact in this cellular context. Our data identify a miRNA profile of myeloid progenitor cells, extending previous information in the field on more primitive stem cells. We further identified changes in the levels of these miRNAs as the cells mature into two separate myeloid lineages. We focused on two miRNAs miR-125b and miR-10a and uncovered their potential role in this process by identifying their likely mRNA targets. We provide biological validation of some of these targets revealing further their specific roles in the development of mature monocytes and granulocytes from the myeloid progenitor.

## Materials and Methods

### Cord blood collection and cell isolation

All research and procedures were performed in accordance with all relevant guidelines and regulations. Human cord blood from healthy donors was obtained from the Sydney Cord Blood Bank following informed consent according to approved protocols (X06-0153 and X09-0111) from the Sydney Local Health District Human Research Ethics Committee. Typically, samples contained 30–60 mL of cord blood with added citrate-dextrose anti-coagulant (ACD-A). Following separation using Ficoll-Paque the mononuclear cells were collected for isolation of monocytes and progenitors. The granulocytes were further isolated by centrifugation and red cell lysis following layering over a solution of 3% (w/v) dextran (Sigma-Aldrich; Sydney, Australia). In some experiments (indicated in text) CD34^+^ cells were enriched from the mononuclear cell fraction using MACS beads and a positive selection kit (Cat # 130-046-702; Miltenyi Biotec), following the manufacturer’s instructions.

### Flow cytometry

All antibodies used for FACS staining were obtained from Biolegend (San Diego, California, USA). The lineage (lin) cocktail comprised antibodies against CD3 (clone HIT3a), CD7 (MEM-186), CD10 (HI10a), CD11b (ICRF44), CD14 (M5E2), CD19 (HIB19) and CD56 (MEM-188). The antibodies against CD34, CD38 and CD235a were from the following clones respectively: 4H11, HIT2 and HIR2. Cells were sorted to >90% purity on either a BD FACS Vantage or FACS Aria. Analytical flow cytometry was performed on a BD FACS Canto.

### Methocult assays

FACS purified myeloid progenitor cells were resuspended in Iscove’s Modified Dulbecco’s Medium (IMDM) then added to methylcellulose containing the cytokines GM-CSF, EPO, interleukin-3 (IL-3) and stem cell factor (SCF) (Methocult, H4434; Stem Cell Technologies) and plated in triplicate into 35 mm Petri dishes (1.1 mL/dish). The plates were incubated at 37 °C, 5% CO_2_ in a humidified environment for 10–16 days. Colony formation was observed using a MZFLIII microscope (Leica Microsystems, Wetzlar, Germany) and photographed with a DFC500 camera attached (Leica).

### TaqMan^®^ Low Density Arrays (TLDA) and miRNA assays

Ten cord blood samples were used for miRNA profiling. MiRNA profiling was performed using TaqMan Low Density Arrays (TLDAs – early access version; Applied Biosystems; Foster City, California, USA). Two TLDAs were performed for each cell type with a pool of 5 samples on each array. RNA was extracted from cells using Trizol reagent (Invitrogen; Sydney, Australia) following the manufacturer’s instructions. A total of 100 ng of RNA was reverse transcribed using Megaplex primers (Human Pool set v1, pre-release evaluation version; Applied Biosystems). The cDNA and master-mix was loaded on to the TLDA card and amplified using an Applied Biosystems 7900HT Fast Real-Time PCR System. The raw data were first analysed using RQ Manager software (version 1.2; Applied Biosystems) and then using Qiagen’s GeneGlobe Data Analysis Centre: (http://pcrdataanalysis.sabiosciences.com/pcr/arrayanalysis.php). Validation of array results was performed using TaqMan miRNA Assays on the 10 samples used for the array individually. All qRT-PCRs performed in this study to amplify specific miRNAs were undertaken using TaqMan miRNA assays specific for the miRNA of interest on a Corbett Rotor Gene (RG 3000; Qiagen, Doncaster, Victoria, Australia).

### Microarrays

Total RNA from myeloid progenitors, monocytes and granulocytes isolated from human cord blood was extracted using TRIzol reagent (Life Technologies) and profiled using Affymetrix GeneChip Gene 1.0 ST human arrays. The raw data were processed using the RMA method from the R Bioconductor ‘oligo’ package for background subtraction, normalization, and summarization of probe level data. They were then plotted as heat maps using the web-based tool Morpheus (software.broadinstitute.org/morpheus).

### Luciferase assays

The sequences of 3′ UTRs used for luciferase reporter assays were PCR-amplified from normal human genomic DNA and sequence verified, then cloned into the dual luciferase vector pSiCHECK-2™ and sequence verified again. To create mutant miRNA binding site vectors, mutagenesis was performed using the enzyme Phusion® (Thermo Fisher). For the assay the plasmids were transfected into HeLa cells using Lipofectamine™2000 (Invitrogen; Carlsbad, CA, USA) according to the manufacturer’s instructions. miRIDIAN hairpin inhibitor control siRNA (IN-001005-01-05) or siRNA targeting miR-125b and miR-10a (IH-300595-05, IH-300549-05 respectively, all from Thermo Scientific Dharmacon) were used to suppress miRNA activity. Transfected cells were incubated for 24 h at 37 °C, 5% CO_2_ followed by lysis. A dual luciferase assay using Dual-Glo Stop & Glo substrate (Promega) was performed on a Victor^2^ Wallac Plate Reader.

## Electronic supplementary material

SUPPLEMENTARY DATA

## References

[1] Akashi K, Traver D, Miyamoto T, Weissman IL (2000). A clonogenic common myeloid progenitor that gives rise to all myeloid lineages. Nature.

[2] Kondo M, Weissman IL, Akashi K (1997). Identification of clonogenic common lymphoid progenitors in mouse bone marrow. Cell.

[3] Majeti R, Park CY, Weissman IL (2007). Identification of a hierarchy of multipotent hematopoietic progenitors in human cord blood. Cell Stem Cell.

[4] Manz MG, Miyamoto T, Akashi K, Weissman IL (2002). Prospective isolation of human clonogenic common myeloid progenitors. Proc Natl Acad Sci USA.

[5] Mori Y, Chen JY, Pluvinage JV, Seita J, Weissman IL (2015). Prospective isolation of human erythroid lineage-committed progenitors. Proc Natl Acad Sci USA.

[6] Fogg DK (2006). A clonogenic bone marrow progenitor specific for macrophages and dendritic cells. Science.

[7] Kawamura S (2017). Identification of a Human Clonogenic Progenitor with Strict Monocyte Differentiation Potential: A Counterpart of Mouse cMoPs. Immunity.

[8] Lee J (2017). Lineage specification of human dendritic cells is marked by IRF8 expression in hematopoietic stem cells and multipotent progenitors. Nat Immunol.

[9] Doulatov S (2010). Revised map of the human progenitor hierarchy shows the origin of macrophages and dendritic cells in early lymphoid development. Nat Immunol.

[10] Villani, A. C. *et al*. Single-cell RNA-seq reveals new types of human blood dendritic cells, monocytes, and progenitors. *Science* **356** (2017). 10.1126/science.aah4573 PMC5775029 28428369

[11] Metcalf D (2008). Hematopoietic cytokines. Blood.

[12] Rosenbauer F, Tenen DG (2007). Transcription factors in myeloid development: balancing differentiation with transformation. Nat Rev Immunol.

[13] Ivey KN, Srivastava D (2015). microRNAs as Developmental Regulators. Cold Spring Harb Perspect Biol.

[14] Bartel DP (2009). MicroRNAs: target recognition and regulatory functions. Cell.

[15] Ritchie W, Rasko JE (2014). Refining microRNA Target Predictions: Sorting the wheat from the chaff. Biochem Biophys Res Commun.

[16] Ebert MS, Sharp PA (2012). Roles for microRNAs in conferring robustness to biological processes. Cell.

[17] Felli N (2005). MicroRNAs 221 and 222 inhibit normal erythropoiesis and erythroleukemic cell growth via kit receptor down-modulation. Proc Natl Acad Sci USA.

[18] Fontana L (2007). MicroRNAs 17-5p-20a-106a control monocytopoiesis through AML1 targeting and M-CSF receptor upregulation. Nat Cell Biol.

[19] Larsen MT (2013). MicroRNA profiling in human neutrophils during bone marrow granulopoiesis and *in vivo* exudation. PLoS ONE.

[20] Cattaneo M (2015). A miRNA Signature in Human Cord Blood Stem and Progenitor Cells as Potential Biomarker of Specific Acute Myeloid Leukemia Subtypes. J Cell Physiol.

[21] Georgantas RW (2007). CD34+ hematopoietic stem-progenitor cell microRNA expression and function: a circuit diagram of differentiation control. Proc Natl Acad Sci USA.

[22] Tenedini E (2010). Integrated analysis of microRNA and mRNA expression profiles in physiological myelopoiesis: role of hsa-mir-299-5p in CD34+ progenitor cells commitment. Cell Death Dis.

[23] Forrest AR (2010). Induction of microRNAs, mir-155, mir-222, mir-424 and mir-503, promotes monocytic differentiation through combinatorial regulation. Leukemia.

[24] Rosa A (2007). The interplay between the master transcription factor PU.1 and miR-424 regulates human monocyte/macrophage differentiation. Proc Natl Acad Sci USA.

[25] Schmeier S (2009). Deciphering the transcriptional circuitry of microRNA genes expressed during human monocytic differentiation. BMC Genomics.

[26] Katzerke C (2013). Transcription factor C/EBPα-induced microRNA-30c inactivates Notch1 during granulopoiesis and is downregulated in acute myeloid leukemia. Blood.

[27] Pulikkan JA (2010). Cell-cycle regulator E2F1 and microRNA-223 comprise an autoregulatory negative feedback loop in acute myeloid leukemia. Blood.

[28] Pulikkan JA (2010). C/EBPα regulated microRNA-34a targets E2F3 during granulopoiesis and is down-regulated in AML with CEBPA mutations. Blood.

[29] Velu CS, Baktula AM, Grimes HL (2009). Gfi1 regulates miR-21 and miR-196b to control myelopoiesis. Blood.

[30] Ghani S (2011). Macrophage development from HSCs requires PU.1-coordinated microRNA expression. Blood.

[31] Vian L (2014). Transcriptional fine-tuning of microRNA-223 levels directs lineage choice of human hematopoietic progenitors. Cell Death Differ.

[32] Merkerova M, Vasikova A, Belickova M, Bruchova H (2010). MicroRNA expression profiles in umbilical cord blood cell lineages. Stem Cells Dev.

[33] Raghavachari N (2014). Integrated analysis of miRNA and mRNA during differentiation of human CD34+ cells delineates the regulatory roles of microRNA in hematopoiesis. Exp Hematol.

[34] Lu J (2008). MicroRNA-mediated control of cell fate in megakaryocyte-erythrocyte progenitors. Dev Cell.

[35] Bissels U (2011). Combined characterization of microRNA and mRNA profiles delineates early differentiation pathways of CD133+ and CD34+ hematopoietic stem and progenitor cells. Stem Cells.

[36] Liao R (2008). MicroRNAs play a role in the development of human hematopoietic stem cells. J Cell Biochem.

[37] Emmrich S (2014). miR-99a/100~125b tricistrons regulate hematopoietic stem and progenitor cell homeostasis by shifting the balance between TGFβ and Wnt signaling. Genes Dev.

[38] Bernstein ID, Andrews RG, Zsebo KM (1991). Recombinant human stem cell factor enhances the formation of colonies by CD34+ and CD34+ lin- cells, and the generation of colony-forming cell progeny from CD34+ lin- cells cultured with interleukin-3, granulocyte colony-stimulating factor, or granulocyte-macrophage colony-stimulating factor. Blood.

[39] Betel D, Wilson M, Gabow A, Marks DS, Sander C (2008). The microRNA.org resource: targets and expression. Nucleic Acids Res.

[40] John B (2004). Human MicroRNA targets. PLoS Biol.

[41] Alemdehy MF, Erkeland SJ (2012). MicroRNAs: key players of normal and malignant myelopoiesis. Curr Opin Hematol.

[42] Gordon JE, Wong JJ, Rasko JE (2013). MicroRNAs in myeloid malignancies. Br J Haematol.

[43] Labbaye C (2008). A three-step pathway comprising PLZF/miR-146a/CXCR4 controls megakaryopoiesis. Nat Cell Biol.

[44] Gerrits A (2012). Genetic screen identifies microRNA cluster 99b/let-7e/125a as a regulator of primitive hematopoietic cells. Blood.

[45] Wang XS, Zhang JW (2008). The microRNAs involved in human myeloid differentiation and myelogenous/myeloblastic leukemia. J Cell Mol Med.

[46] Guglielmelli P (2011). Overexpression of microRNA-16-2 contributes to the abnormal erythropoiesis in polycythemia vera. Blood.

[47] Fazi F (2005). A minicircuitry comprised of microRNA-223 and transcription factors NFI-A and C/EBPalpha regulates human granulopoiesis. Cell.

[48] Johnnidis JB (2008). Regulation of progenitor cell proliferation and granulocyte function by microRNA-223. Nature.

[49] Felli N (2009). MicroRNA 223-dependent expression of LMO2 regulates normal erythropoiesis. Haematologica.

[50] Fellenberg J (2016). Restoration of miR-127-3p and miR-376a-3p counteracts the neoplastic phenotype of giant cell tumor of bone derived stromal cells by targeting COA1, GLE1 and PDIA6. Cancer Lett.

[51] Ye FG (2015). Cytidine Deaminase Axis Modulated by miR-484 Differentially Regulates Cell Proliferation and Chemoresistance in Breast Cancer. Cancer Res.

[52] Yu Y (2016). MicroRNA-127 is aberrantly downregulated and acted as a functional tumor suppressor in human pancreatic cancer. Tumour Biol.

[53] Heike T, Nakahata T (2002). *Ex vivo* expansion of hematopoietic stem cells by cytokines. Biochim Biophys Acta.

[54] Shaham L, Binder V, Gefen N, Borkhardt A, Izraeli S (2012). MiR-125 in normal and malignant hematopoiesis. Leukemia.

[55] Bousquet M (2008). Myeloid cell differentiation arrest by miR-125b-1 in myelodysplastic syndrome and acute myeloid leukemia with the t(2;11)(p21; q23) translocation. J Exp Med.

[56] Bousquet M, Harris MH, Zhou B, Lodish HF (2010). MicroRNA miR-125b causes leukemia. Proc Natl Acad Sci USA.

[57] O’Connell RM (2010). MicroRNAs enriched in hematopoietic stem cells differentially regulate long-term hematopoietic output. Proc Natl Acad Sci USA.

[58] Ooi AG (2010). MicroRNA-125b expands hematopoietic stem cells and enriches for the lymphoid-balanced and lymphoid-biased subsets. Proc Natl Acad Sci USA.

[59] Wong JJ (2014). Identification of nuclear-enriched miRNAs during mouse granulopoiesis. J Hematol Oncol.

[60] Bousquet M, Nguyen D, Chen C, Shields L, Lodish HF (2012). MicroRNA-125b transforms myeloid cell lines by repressing multiple mRNA. Haematologica.

[61] Surdziel E (2011). Enforced expression of miR-125b affects myelopoiesis by targeting multiple signaling pathways. Blood.

[62] Sun SM (2011). Transition of highly specific microRNA expression patterns in association with discrete maturation stages of human granulopoiesis. Br J Haematol.

[63] Ritchie W, Flamant S, Rasko JE (2009). Predicting microRNA targets and functions: traps for the unwary. Nat Methods.

[64] Nakayama F (2001). CD15 expression in mature granulocytes is determined by alpha 1,3-fucosyltransferase IX, but in promyelocytes and monocytes by alpha 1,3-fucosyltransferase IV. J Biol Chem.

[65] Buffone A (2013). Silencing α1,3-fucosyltransferases in human leukocytes reveals a role for FUT9 enzyme during E-selectin-mediated cell adhesion. J Biol Chem.

[66] Martinez M (2005). Regulation of PSGL-1 interactions with L-selectin, P-selectin, and E-selectin: role of human fucosyltransferase-IV and -VII. J Biol Chem.

[67] Dagia NM (2006). G-CSF induces E-selectin ligand expression on human myeloid cells. Nat Med.

[68] Jia HY (2012). MicroRNA-125b functions as a tumor suppressor in hepatocellular carcinoma cells. Int J Mol Sci.

[69] Wu S (2015). miR-125b Suppresses Proliferation and Invasion by Targeting MCL1 in Gastric Cancer. Biomed Res Int.

[70] Zeng CW (2012). Camptothecin induces apoptosis in cancer cells via microRNA-125b-mediated mitochondrial pathways. Mol Pharmacol.

[71] Dzhagalov I, St John A, He YW (2007). The antiapoptotic protein Mcl-1 is essential for the survival of neutrophils but not macrophages. Blood.

[72] Steimer DA (2009). Selective roles for antiapoptotic MCL-1 during granulocyte development and macrophage effector function. Blood.

[73] Rossi RL (2011). Distinct microRNA signatures in human lymphocyte subsets and enforcement of the naive state in CD4+ T cells by the microRNA miR-125b. Nat Immunol.

[74] Chaudhuri AA (2012). Oncomir miR-125b regulates hematopoiesis by targeting the gene Lin28A. Proc Natl Acad Sci USA.

[75] Garzon R (2006). MicroRNA fingerprints during human megakaryocytopoiesis. Proc Natl Acad Sci USA.

[76] Zarif MN (2013). Megakaryocytic differentiation of CD133+ hematopoietic stem cells by down-regulation of microRNA-10a. Hematology.

[77] Feinberg MW (2007). The Kruppel-like factor KLF4 is a critical regulator of monocyte differentiation. EMBO J.

[78] Bryant A (2012). miR-10a is aberrantly overexpressed in Nucleophosmin1 mutated acute myeloid leukaemia and its suppression induces cell death. Mol Cancer.

[79] Takahashi H (2012). TGF-β and retinoic acid induce the microRNA miR-10a, which targets Bcl-6 and constrains the plasticity of helper T cells. Nat Immunol.

[80] Agirre X (2008). Down-regulation of hsa-miR-10a in chronic myeloid leukemia CD34+ cells increases USF2-mediated cell growth. Mol Cancer Res.

