# Supplementary material for: Identifying microRNA determinants of human myelopoiesis
Source: Sci Rep. 2018 May 8;8:7264. doi: 10.1038/s41598-018-24203-7 (PMC5940821; doi:10.1038/s41598-018-24203-7)
Supplement: Supplementary file 1 — SUPPLEMENTARY DATA [file 41598_2018_24203_MOESM1_ESM.pdf]

Identifying microRNA determinants of human myelopoiesis

Authors:

Megha Rajasekhar, Ulf Schmitz, Stephane Flamant, Justin JL Wong, Charles G Bailey, William Ritchie, Jeff Holst, John EJ Rasko

Supplementary Information:

a

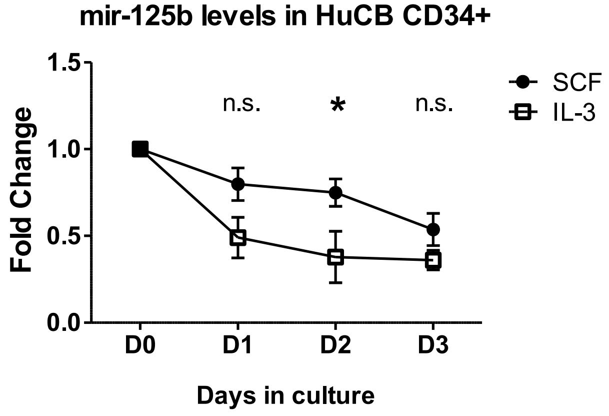

b

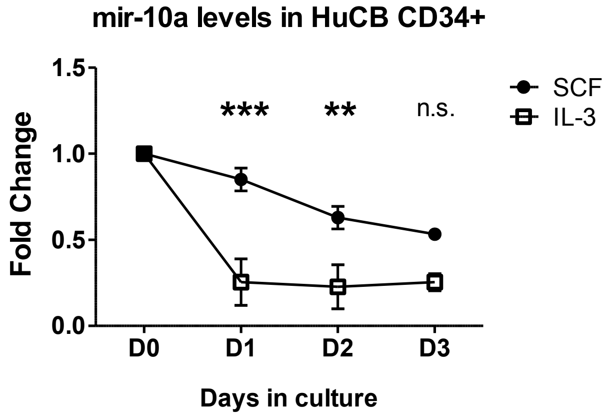

Supplementary Figure 1. The effect of the cytokines stem cell factor (SCF) and interleukin-3 (IL-3) on levels of miR-125b and miR-10a. FACS sorted human cord blood CD34+ cells were cultured in medium with serum and added SCF (100 ng/mL) or IL-3 (100 ng/mL) for three days. Cells were collected every day and levels of (a) miR-125b and (b) miR-10a were assessed by Taqman miRNA assays and normalised first to RNU24 and then to the values *ex vivo* (D0) to give fold change. Data were tested by two-way ANOVA with Bonferroni post-test (results shown on graphs; *** $p<0.001$, **$p<0.01$, *$p<0.05$, n.s.=not significant). Both the cytokine and time had a significant effect on levels of mir-125b ($p=0.0032$ and $p<0.0001$ respectively) and on levels of mir-10a ($p<0.0001$ and $p<0.0001$ respectively).

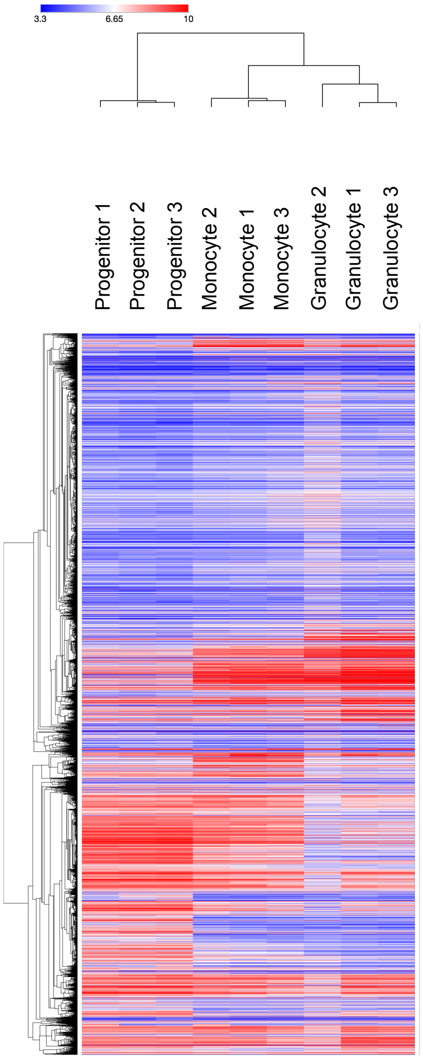

Supplementary Figure 2. Gene expression profiles of myeloid progenitors, monocytes and granulocytes. Affymetrix gene expression profiling was conducted on flow sorted, cord blood myeloid progenitors, monocytes and granulocytes from three samples. Transformed (log 2) values from the full dataset of 19502 transcripts was used for unsupervised hierarchical clustering.

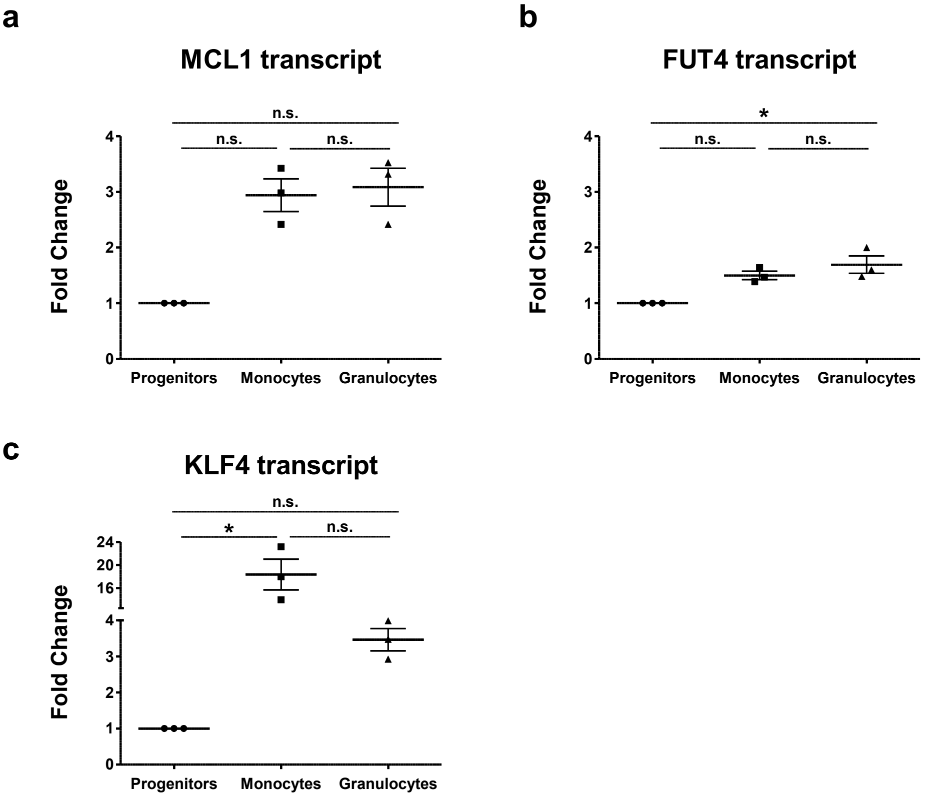

Supplementary Figure 3. Expression profiles of predicted miRNA targets in myeloid progenitors, monocytes and granulocytes. Affymetrix gene expression profiling was conducted on flow sorted, cord blood myeloid progenitors, monocytes and granulocytes from three samples. Expression profiles of predicted targets of miR-125b (a,b) and miR-10a (c) are shown as fold change in monocytes and granulocytes relative to progenitors. The groups were tested by one-way ANOVA with Kruskal-Wallis test and Dunn's post test. * p<0.05.

a

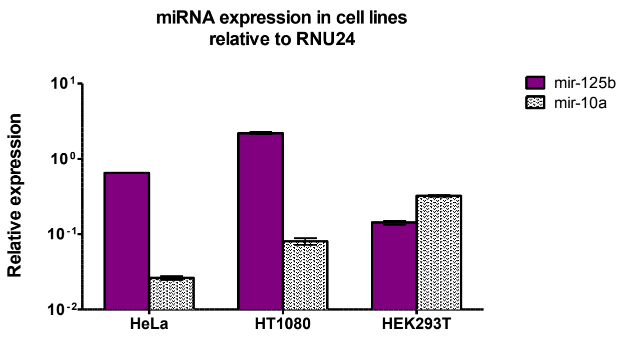

b

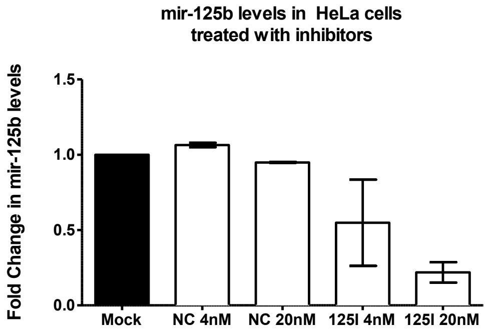

Supplementary Figure 4. Testing miRNA levels in cell lines. (a) Levels of endogenous miR-125b and miR-10a in three easily transfectable cell lines; normalised to RNU24. (b) The inhibitor for miR-125b (125I) or negative control inhibitor (Neg C) was transfected into HeLa cells at concentrations of 4 or 20 nM and levels of miR-125b measured by RT-qPCR after overnight culture in order to confirm effective knockdown of the miRNA.
